# An Antifungal Chitosanase from *Bacillus subtilis* SH21

**DOI:** 10.3390/molecules26071863

**Published:** 2021-03-25

**Authors:** Yuanxiang Pang, Jianjun Yang, Xinyue Chen, Yu Jia, Tong Li, Junhua Jin, Hui Liu, Linshu Jiang, Yanling Hao, Hongxing Zhang, Yuanhong Xie

**Affiliations:** 1 Key Laboratory of Agricultural Product Detection and Control of Spoilage Organisms and Pesticides, Beijing Laboratory for Food Quality and Safety, Beijing Engineering Laboratory of Probiotics Key Technology Development, Beijing Engineering Technology Research Center of Food Safety Immune Rapid Detection, Food Science and Engineering College, Beijing University of Agriculture, Beijing 102206, China; pyx17801206852@163.com (Y.P.); 18811626398@163.com (J.Y.); 13651064909@163.com (X.C.); jiayu11003@163.com (Y.J.); leetong0606@163.com (T.L.); jinjunhua002008@163.com (J.J.); mxdlh1963@163.com (H.L.); jls@bua.edu.cn (L.J.); 2 Key Laboratory of Functional Dairy Science of Beijing and Chinese Ministry of Education, College of Food Science and Nutritional Engineering, China Agricultural University, Beijing 100083, China; haoyl@cau.edu.cn

**Keywords:** *Bacillus subtilis*, antifungal chitosanase, *Fusarium solani*, structure analysis

## Abstract

*Bacillus subtilis* SH21 was observed to produce an antifungal protein that inhibited the growth of *F. solani*. To purify this protein, ammonium sulfate precipitation, gel filtration chromatography, and ion-exchange chromatography were used. The purity of the purified product was 91.33% according to high-performance liquid chromatography results. Sodium dodecyl sulfate–polyacrylamide gel electrophoresis and liquid chromatography–tandem mass spectrometry (LC–MS/MS) analysis revealed that the molecular weight of the protein is 30.72 kDa. The results of the LC–MS/MS analysis and a subsequent sequence-database search indicated that this protein was a chitosanase, and thus, we named it chitosanase SH21. Scanning and transmission electron microscopy revealed that chitosanase SH21 appeared to inhibit the growth of *F. solani* by causing hyphal ablation, distortion, or abnormalities, and cell-wall depression. The minimum inhibitory concentration of chitosanase SH21 against *F. solani* was 68 µg/mL. Subsequently, the corresponding gene was cloned and sequenced, and sequence analysis indicated an open reading frame of 831 bp. The predicted secondary structure indicated that chitosanase SH21 has a typical a-helix from the glycoside hydrolase (GH) 46 family. The tertiary structure shared 40% similarity with that of *Streptomyces sp.* N174. This study provides a theoretical basis for a topical cream against fungal infections in agriculture and a selection marker on fungi.

## 1. Introduction

Diseases caused by *Fusarium solani* are a limiting factor in plant production and yield quantity. *F. solani* causes the death of young and adult plants, with consequent economic losses [1]. *Bacillus subtilis* has been recognized as a promising biocontrol agent against various fungal phytopathogens [2,3]. This bacterium produces several antifungal proteins, including chitosanases [4,5,6], in addition to volatile compounds [7] and antifungal lipopeptides [8,9]. Thus, it is clear that *Bacillus subtilis* is a source of substances that may be useful for protecting plants from phytopathogens.

Chitosan is a deacetylated product of chitin, which is a long-chain polymer of *N*-acetylglucosamine [10]. Chitosanases (EC 3.2.1.132) catalyze the hydrolysis of the β-1,4-linked glycosidic linkages of chitosan [11]. According to previous reports, chitosanases have been found in various microorganisms, including fungi [12,13,14] and bacteria [15,16,17,18]. Based on the differences in the primary structure, chitosanases are classified into six glycoside hydrolase (GH) families, namely GH5, GH7, GH8, GH46, GH75, and GH80 [19]. Chitosanases from different biological sources differ in action mechanism; bacterial chitosanases are often grouped into GH46, whereas fungal chitosanases mostly belong to GH75 [20].

Chitosanases can be used for the biotransformation of chitosan and biocontrol of phytopathogenic fungi [21]. So far, chitosanases from microorganisms have been widely used against phytopathogenic fungi. The pesticidal activity of each chitosanase against plant pathogenic fungi varies due to the differences in chitin content and degree of exposure to the glycosidic chains in the chitin of the cell wall among different phytopathogenic fungi [22,23,24], as well as the immune response of the plant. This study aimed to identify and purify a *Bacillus* SH21 antifungal protein exhibiting a fungistatic activity against *F. solani* and to evaluate the underlying action mechanism.

## 2. Results

### 2.1. Identification of Bacillus SH21

Microbiological characterization revealed that *Bacillus sp.* SH21 isolated from a paste of soybeans is a typical rod-shaped, catalase-positive, spore-forming, and aerobic bacterium. By homology analysis in the GenBank nucleotide database, the 16S rDNA (Deposit in Genbank: MN756647) of *Bacillus sp.* SH21 was found to be 100% identical to the corresponding 16S rDNAs of *B. subtilis* (Genbank: MK860024 and KP717559) in the database. Thus, we named this strain *B. subtilis* SH21 and deposited it at the China General Microbiological Culture Collection Center (CGMCC 18612).

### 2.2. Purification of the Antifungal Protein from B. subtilis SH21

**Figure 1:**
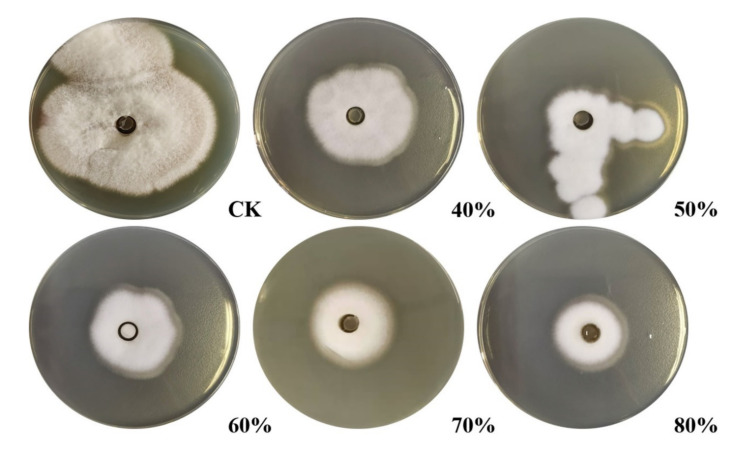
Inhibitory effects of (NH_4_)_2_SO_4_-saturated crude protein fractions on *F. solani* (0–40%, 0–50%, 0–60%, 0–70%, 0–80%). Check the Tris-HCL instead of crude protein. The crude protein was dialyzed after (NH_4_)_2_SO_4_ precipitation, and the dialyzed procedure as follows: Dialysis external fluid, Tris-HCl; dialysis time, 24 h.

**Figure 2:**
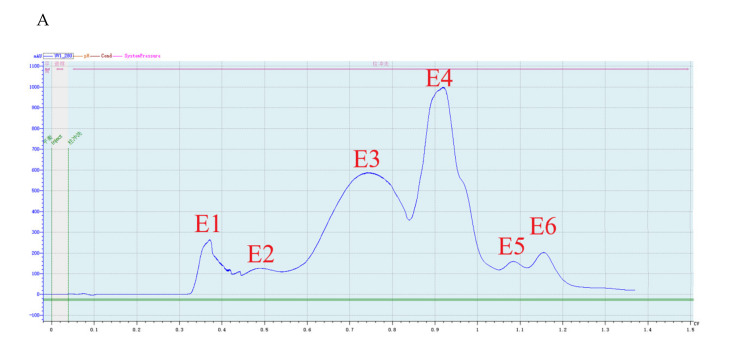

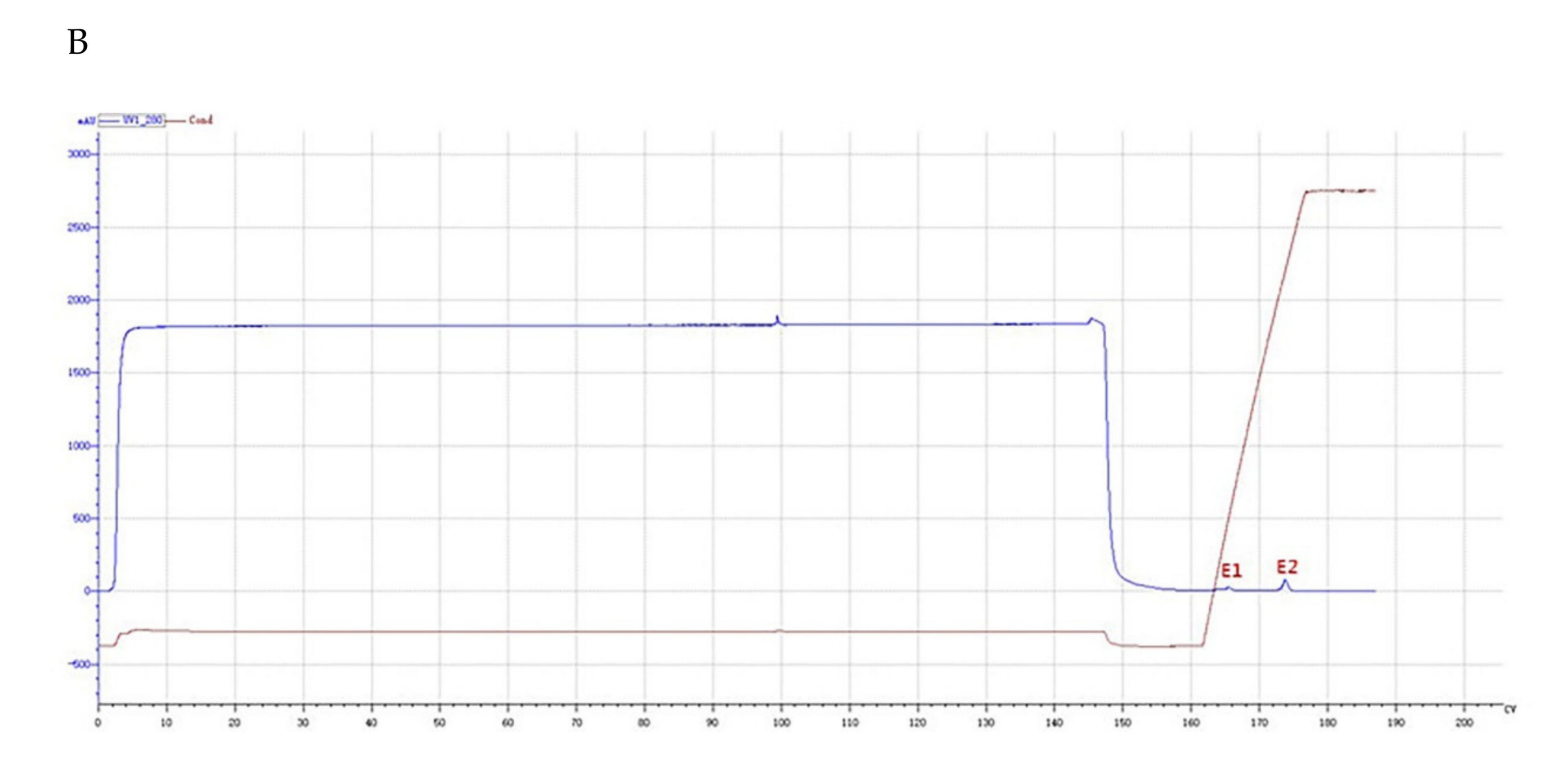
Purification of the antifungal protein. (**A**) Fractionation via Superdex75PG gel filtration chromatography. The volume which was loaded onto Superdex 75 column was 5 mL. E1 and E2 peaks were not collected. E3 was 15 mL, E4 was 25 mL, E5 was 7 mL, and E6 was 11.5 mL. (**B**) Fractionation via SP Focurose HPR ion-exchange chromatography. The volume of fraction E1 was 5 mL. The volume of fraction E2 was 7.5 mL.

**Figure 3:**
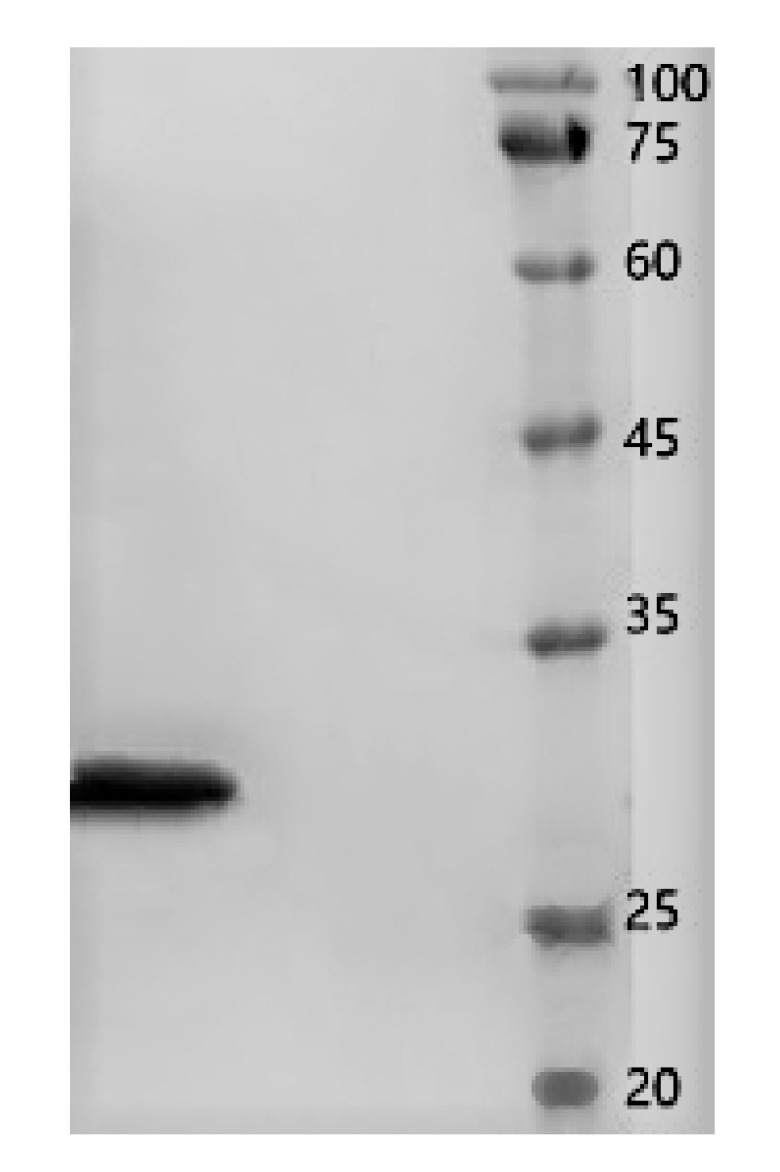
SDS-PAGE results. Left lane, the purified antifungal protein from *B. subtilis* SH21; right lane, molecular-weight markers.

Crude protein from *B. subtilis* SH21 culture was precipitated using (NH_4_)_2_SO_4_ solutions with various saturation levels. The greatest inhibitory effect was exhibited by the precipitate in the 80% saturated solution (Figure 1). Then, the dialyzed precipitate was subjected to Superdex75PG gel filtration chromatography, which furnished an elution profile with six peaks (Figure 2A). Among the six different fractions eluted from the gel matrix, peak E4 had the highest antifungal activity. Fraction E4 was further purified via ion-exchange chromatography, and two peaks were subsequently eluted from this fraction (Figure 2B). Peak E1 showed the highest antifungal activity. The yield of the antifungal protein after each purification procedure was shown in Table 1, and the recovery rate was 1.2% at the end. The homogeneity of the purified protein was ascertained using SDS-PAGE (Figure 3). A single protein band was observed at approximately 30 kDa.

### 2.3. Assessment of the Protein Purity

**Figure 4:**
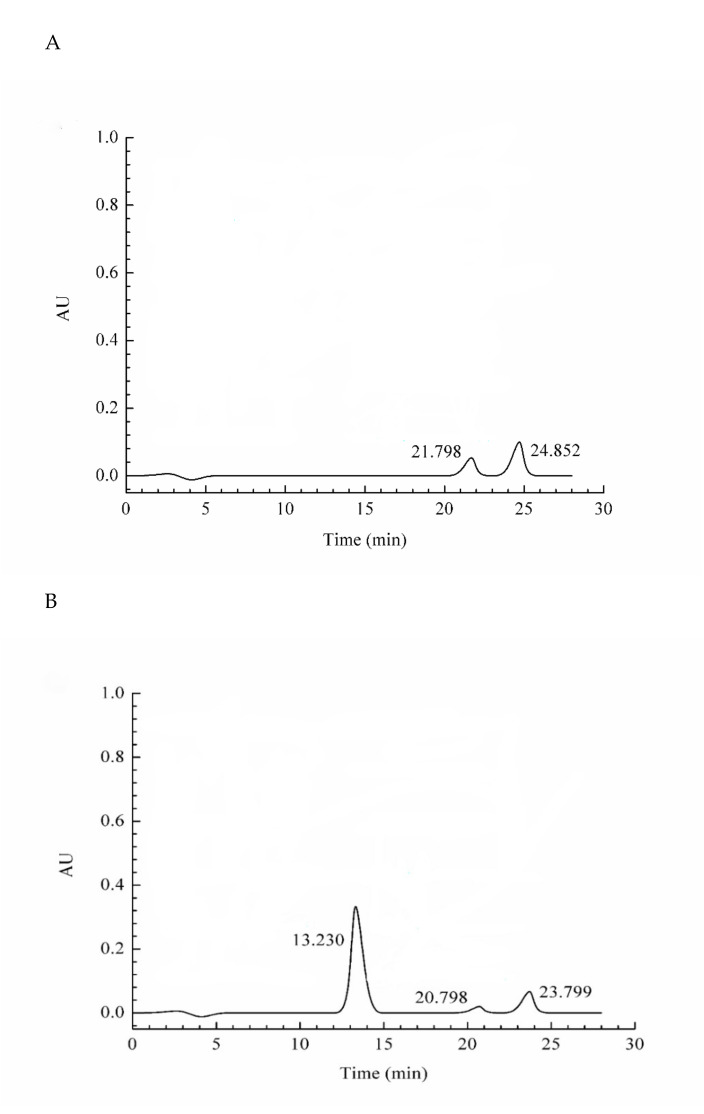
The purity of the purified antifungal protein. (**A**) The blank used phosphate-buffered saline (PBS) instead of purified antifungal protein, (**B**) The purified antifungal protein. The peak areas were 1,395,873, 48,080, and 84,454 at 13.230 min, 20.798 min, and 23.799 min, respectively. The purity was expressed by the ratio of each peak area to all peak areas.

The purity of purified protein was measured by HPLC. Currently, HPLC has become an important tool for separating and detecting proteins due to its fast analysis speed and good sensitivity. The purity of the purified antifungal protein was shown in Figure 4. Three absorption peaks were detected at 13.230, 20.798, and 23.799 min. The absorption peak at 13.230 min was identified to be the target protein, and its purity reached 91.33%.

### 2.4. Characterization of the Protein via LC–MS/MS Analysis

The amino acid sequence of the purified antifungal protein was determined via LC–MS/MS. The protein pattern was closest to that of a chitosanase (D4FZ80). A high Mowse score of 323.31 (*p* < 0.05) was obtained. The sequence coverage was 86.3%, and the exact molecular mass of the protein was 30.72 kDa (Table 2). These results indicated that the antifungal protein purified from *B. subtilis* SH21 was a chitosanase, and thus, we named it chitosanase SH21.

### 2.5. Assessment of the Minimum Inhibitory Concentration of Chitosanase SH21 Against F. solani 

**Figure 5:**
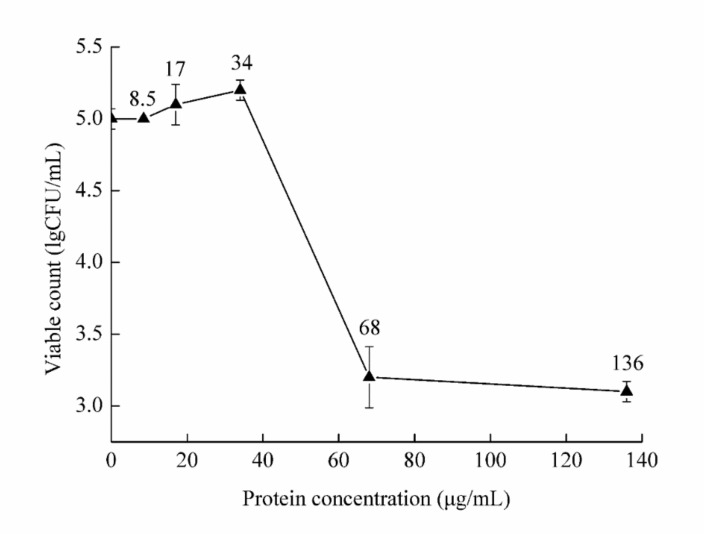
The minimum inhibitory concentration of chitosanase SH21 against *F. solani.*

*F. solani* culture was treated with the protein at five concentrations. The viable count was then determined and compared with that of the control treatment (Figure 5). The minimum inhibitory concentration (MIC) value of chitosanase SH21 against *F. solani* was estimated at 68 µg/mL.

### 2.6. SEM and TEM Assessment of the Effect of Chitosanase SH21 on F. solani

**Figure 6:**
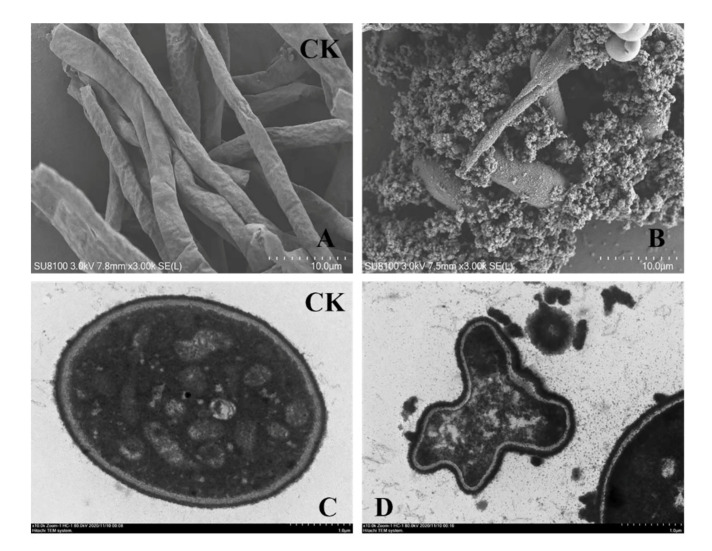
Effects of chitosanase SH21 on *F. solani*. (**A**) Hyphae of untreated *F. solani*, (**B**) Hyphae of *F. solani* treated with chitosanase SH21 for 24 h (Bar = 10.0 µm). (**C**) Cell wall of untreated *F. solani*, (**D**) Abnormal *F. solani* cells upon chitosanase SH21 treatment for 24 h (Bar = 1.0 µm).

SEM studies revealed severe morphological alterations in *F. solani* hyphae after a 24 h culture with chitosanase SH21. Compared with no treatment (Figure 6A), chitosanase SH21 evoked hyphal distortion or ablation, and protoplasm extravasation (Figure 6B). TEM results showed that chitosanase SH21 had a severe impact on the ultrastructure of *F. solani* (Figure 6C), including cell wall depression and thickening, and cytoplasmic disintegration (Figure 6D). Therefore, we concluded that chitosanase SH21 interfered with *F. solani* hyphal metabolism, causing the leakage of intracellular substances and the accumulation and deposition of protoplasts. Subsequently, the leakage of intracellular substances further caused cell metabolism disorders, leading to a series of cellular structural changes, ultimately inhibiting the growth of *F. solani*.

### 2.7. Sequence, Structure Analysis, and Modeling

The chitosanase SH21 gene was cloned. An entire open reading frame (ORF)encoding a potential chitosanolytic gene was identified, consisting of 831 bp and encoding a protein of 277 amino acid residues. The nucleotide sequence of the chitosanolytic gene has been submitted to the GenBank database with accession number MW132599. Signal peptide analysis shows that chitosanase SH21 contains a putative signal peptide (Met1 to Phe36). According to a search of the Conserved Domain Database (CDD) of national center for biotechnology information (NCBI), chitosanase SH21 is presumed to be a chitosanase belonging to the glycosyl hydrolase 46 family.

**Figure 7:**
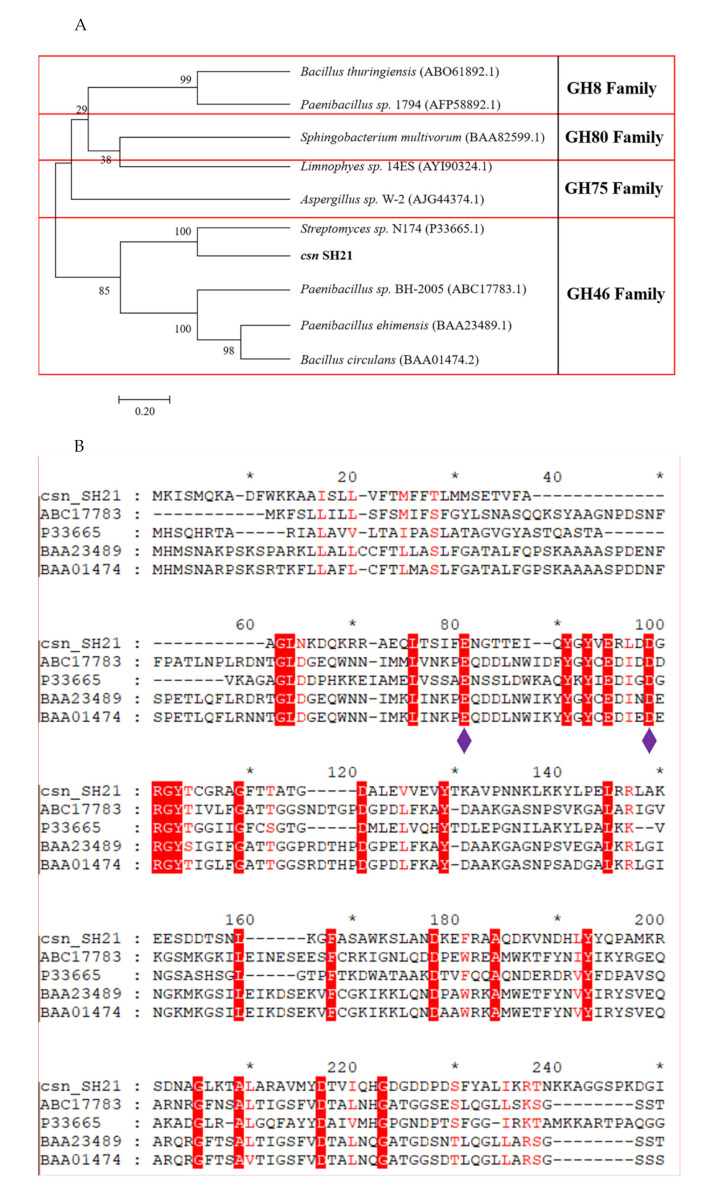

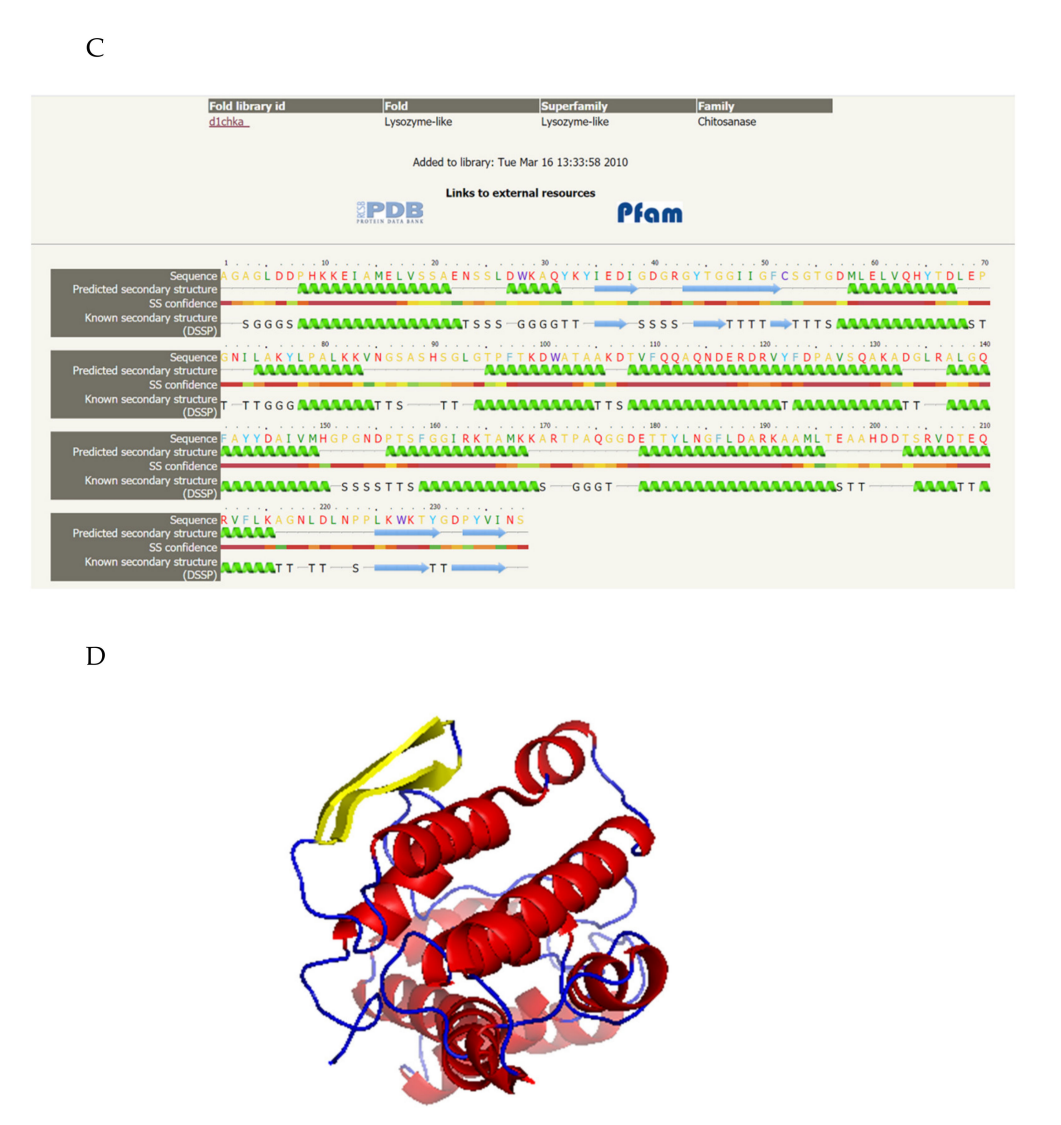
Bioinformatic analysis of csn SH21. (**A**) Neighbor-joining phylogenetic tree. Phylogenetic analysis was carried out using MEGA 6.0 software. Chitosanases in different glycoside hydrolase families are in separate red boxes. (**B**) Multiple amino acid sequence alignment of csn SH21 with chitosanases belonging to glycoside hydrolase family 46. The two catalytic residues Glu81 and Asp99 were marked by purple diamond. (**C**) The predicted secondary structure of csn SH21. The secondary structure analysis was carried out using Phyre2 software. (**D**) 3D-model of csn SH21. The 3D-structure analysis was carried out using Phyre2 software. The a-helix was red, the beta sheet was yellow. The program was carried out using PYMOL.

A phylogenetic tree was created based on the sequences of chitosanase SH21 and other reported chitosanases from glycoside hydrolase (GH) families 8, 80, 75, and 46 (Figure 7A). The phylogenetic tree indicates that the amino acid sequence of csn SH21 is highly similar with a GH family 46 chitosanase 1CHKA (Genbank number: P33665.1) from *Streptomyces sp.* N174. To further explore the structure of csn SH21, a multiple sequence alignment was established among csn SH21 and other four chitosanases which is similar to csn SH21. According to the multiple sequence alignment (Figure 7B), the two conserved glutamic acid (Glu81) and aspartic acid (Asp99) residues were considered as the catalytic residues in csn SH21, playing a key role in catalytic action [25].

The secondary structure and the 3D-model of csn SH21 was built using the Phyre2 server. Using the chitosanase 1CHKA from *Streptomyces sp.* N174 as the template, the predicted secondary structure revealed that csn SH21 had a typical a-helix structure (Figure 7C). As for the 3D-model, which owns the 40% identity to csn SH21 (Figure 7D).

## 3. Discussion

To date, many chitosanases from diverse organisms have been purified and characterized [18,26,27]. The molecular weights of chitosanases differ depending on the enzyme type. Most chitosanases have a low apparent molecular mass within the range of 20–45 kDa. Chitosanases belonging to the GH46 and GH75 families are approximately 30 kDa, whereas the molecular weights of those of the GH8 family are >40 kDa [28]. The molecular weight of a chitosanase from *Bacillus sp.* has been reported to be 30.35 kDa [29], whereas that of the S65 chitosanase from the same source has been estimated to be 45 kDa [30]. The molecular weight of the chitosanase from *B. subtilis* SH21 was similar to those of Csn21c (29.6 kDa) [31], CsnB (30.89 kDa) [15], and CH2 chitosanase (29 kDa) [32], which are chitosanases from *Streptomyces albolongus* ATCC27414, *Bacillus sp.*, and *B. subtilis* CH2, respectively. Amino acid sequencing via Edman degradation has shown that CH2 chitosanase has the 15 AGLNKDQKRRAEQLT amino acid sequence on the N- terminus, and this pattern is similarly found in other chitosanases from *Bacillus* species [32]. However, chitosanase SH21 sequence coverage was 86.3% according to the LC-MS/MS results. Additionally, only few reports have analyzed the purity of the proteins they purified. The purity of chitosanase SH21, identified and purified in this study, reached 91.33% according to the HPLC results.

Chitosanases produced from bacteria and fungi exert significant antifungal activities. Gu et al. have reported that the chitosanase SaChi19B from *Streptomyces alfalae* exerts various inhibitory effects on six plant pathogenic fungi, especially *Botrytis cinereal* and *Rhizoctonia solani* [33]. Song et al. have isolated a chitosanase from *Pedobacter sp.* PR-M6. This chitosanase inhibits the growth of *A. brassicicola, B. cinerea, F. solani,* and *R. solani*, and also severely impacts the germination of their hyphae and spores [34]. All the above-mentioned chitosanases have broad-spectrum antifungal effects. Conversely, chitosanase SH21 has a narrow-spectrum antifungal effect (data not shown).

Many chitosanase genes have been cloned from various microbes. Recently, a chitosanase gene from *B. amyloliquefaciens* YX-01 has been cloned, and the full-length of this gene (BaCsn46A) has an open reading frame of 843 bp, encoding 280 amino acids [16]. The chitosanase gene csn has been cloned from *Penicillium sp.* D-1, and the deduced CSN protein consists of 250 amino acids, including a 20-amino acid signal peptide [35]. In the study presented here, the chitosanase SH21 gene was estimated to have an open reading frame of 831 bp, encoding 277 amino acids, including a 36-amino acid signal peptide. Further research is needed to understand the relationship between the structure and antifungal activity of chitosanase SH21.

## 4. Materials and Methods

### 4.1. Microorganisms, Culture Conditions, and Reagents

*B. subtilis* SH21 was isolated from a paste of soybeans harvested in Sichuan Province, China. It was grown in Luria–Bertani (LB) medium (Solarbio, Beijing, China; 1 L distilled water containing 10 g tryptone, 5 g yeast extract, 10 g NaCl) at 30 °C and fermented in the fermentation medium (1 L distilled water containing 2 g maltose, 2 g casein, 0.05 g FeSO_4_·7H_2_O, 0.5 g MgSO_4_·7H_2_O, 0.7g KH_2_PO_4_, and 0.3 g K_2_HPO_4_) at the same temperature for 48 h. Bacteria were kept frozen in 50% (*v*/*v*) glycerol at −80 °C.

*F. solani* was purchased from China Center of Industrial Culture Collection (CICC2603)*. F. solani* was statically cultured on potato dextrose agar (PDA) at 28 °C for 3 days and then stored at 4 °C.

### 4.2. Strain Identification

The genomic DNA of *B. subtilis* SH21 was extracted using the TIANamp Bacteria DNA Kit, following the manufacturer’s instructions (Tiangen Biotech CO. LTD, Beijing, China). The 16s rDNA nucleotide sequences were amplified via polymerase chain reaction (PCR). The reaction mixture was composed of 9.5 µL of double-distilled H_2_O, 12.5 µL of Ex Taq (Takara Biotech CO.LTD, Dalian, China), 1 µL of each primer (0.5 µM) (27F: 5′-AGAGTTTGATCCTGGCTCAG-3′ and 1492R: 5′-GGTTACCTTGTTACGACTT-3′), and 1 µL of genomic DNA. The PCR program was as follows: 95 °C for 5 min, followed by 34 cycles of 95 °C for 30 s, 55 °C for 30 s, and 72 °C for 30 s. The final extension step was performed at 72 °C for 5 min. Following amplification, the amplified products were purified and sequenced (Sangon Biotech Co. Ltd., Shanghai, China). The nucleotide sequences were matched with sequences in the GenBank database by using the online NCBI BLAST program.

### 4.3. Purification of the Antifungal Protein 

A single colony of *B. subtilis* SH21 was cultured in LB liquid medium at 30 °C for 12 h with shaking at 180 rpm. The resulting seed culture was inoculated into the fermentation medium (2% *v*/*v* ratio) and cultured at 30 °C for 48 h with shaking at 180 rpm. Afterward, the supernatant was collected via centrifugation at 10,000 rpm and 4 °C for 10 min. The supernatant was filtered through a 0.22-µm filter (Millipore, Burlington, MA, USA).

The crude enzyme was precipitated from the supernatant via ammonium sulfate saturation (0–40%, 0–50%, 0–60%, 0–70%, 0–80% saturation) and dissolved in buffer A (0.02 M Tris-HCl, pH 7.8, Nanjing BioChannel Biotechnology Co., Ltd, Nanjing, Jiangsu, China). After dialysis against buffer A, the enzyme dialysate was applied to a Superdex75PG column (1.6 × 64.6 cm, Beijing Huideyi Technology Co., Ltd, Beijing, China) pre-equilibrated with buffer A and eluted using buffer A at a flow rate of 1 mL/min. Each fraction was collected and tested for antifungal activity against *F. solani*. The active fraction exhibiting the maximum antifungal activity was further purified by ion exchange chromatography. This fraction was loaded on an SP Focurose HPR column (Beijing Huiyan Biotechnology Co., Ltd, Beijing, China) previously equilibrated with phosphate-buffered saline (PBS, pH 6.5), and then the bound portion was eluted using PBS (pH 6.5) with a linear gradient from 0 to 1.0 mol/L NaCl at a flow rate of 2.5 mL/min. The eluted fractions were pooled and tested for the antifungal activity.

### 4.4. Determination of the Purity 

The purity of the derived antimicrobial protein was determined via high-performance liquid chromatography (HPLC) with an Ultimate^®^ XB-C4 column (250 × 4.6 mm, 5 μm (Tongpu Experimental Instrument Co. Ltd., Guangzhou, China). The solvents that constituted the mobile phase were (A) 0.1% trifluoroacetic acid and (B) acetonitrile. The elution conditions were as follows: 0–1 min, 0–5% linear gradient of B; 1–25 min, 5–95% linear gradient of B; and 25–28 min, 95–5% linear gradient of B. The flow rate and injection volume were set at 1 mL/min and 10 µL, respectively. The system was operated at 25 °C, and the purity was assessed by measuring the absorbance at 214 nm.

### 4.5. Determination of the Molecular Weight and Amino Acid Sequence 

Protein concentrations were measured using Bradford’s method [36]. The apparent purity and molecular weight (MW) of the antimicrobial protein were assessed via sodium dodecyl sulfate–polyacrylamide gel electrophoresis (SDS-PAGE) using 4% and 12% acrylamide in the stacking and separating gels, respectively [37]. The protein bands were visualized via Coomassie R-250 (Solarbio, Beijing, China) brilliant blue staining.

The molecular mass and amino acid sequence of the purified protein, referred to as chitosanase SH21 henceforth, were determined via liquid chromatography–tandem mass spectrometry (LC–MS/MS) [38]. The fragment spectra were searched against the NCBI non-redundant protein database. The database searches were carried out using the Maxquant search engine. 

### 4.6. Assessment of the Antifungal Activity 

The purified chitosanase SH21 was filtered through sterile 0.22-μm Millipore filters. The inhibitory effects on *F. solani* were assessed using the method of Huang et al. [39], with some modifications. Briefly, 1 mL of protein was first mixed with 20 mL of PDA medium (45 °C, Solarbio, Beijing, China), and then the mix was spread on 90 mm plates. An Oxford cup was vertically placed on the plate, and then 200 μL of the fungal culture was dispensed into the Oxford cup and allowed to diffuse at 4 °C for 2 h. The control experiment was conducted using the PDA medium with PBS instead of protein. All the plates were incubated at 28 °C for 3 days and then assessed for fungal colonies.

### 4.7. The Minimum Inhibitory Concentration (MIC) of the Chitosanase SH21 Against F. solani

The MIC value was measured using the half-dilution method [40]. Briefly, purified chitosanase SH21 was diluted to final concentrations of 8.5, 17, 34, 68, and 136 µg/mL. The diluted samples were then mixed with *F. solani* culture (1.1 × 10^3^ CFU/mL), and each mix was incubated at 28 °C for 24 h with shaking at 180 rpm. PBS instead of protein was used as a negative control. The minimum concentration with no indication of fungal growth was considered the MIC value of chitosanase SH21 against *F. solani*. Each experiment was repeated three times.

### 4.8. Electron Microscopy

The purified chitosanase SH21 was mixed with *F. solani* culture and incubated at 28 °C for 1 day with shaking at 180 rpm. Transmission and scanning electron microscopy (TEM and SEM) samples were prepared as described by Kang [41]. The morphological alterations and ultrastructure of the samples were observed using a SU8100 scanning electron microscope (HITACHI, Tokyo, Japan) and HT7800 transmission electron microscope (HITACHI, Tokyo, Japan), respectively.

### 4.9. Cloning of Chitosanase Gene, Sequence Analysis, and Homology Modeling

The coding sequence of chitosanase SH21 was amplified from the genomic DNA of *B. subtilis* SH21 via PCR using the primers 5′-CATTGTAATCTTGCATGGCTTTAAA-3′ (F) and 5′-TAATGAAAGTTGAAGCAGTATGAAA-3′ (R). The PCR reaction mixture was composed of 9.5 µL of double-distilled H_2_O, 12.5 µL of Ex Taq, 1 µL of each primer (0.5 µM), and 1 µL of genomic DNA. The PCR program was set at 95 °C for 5 min, followed by 30 cycles of 95 °C for 30 s, 52 °C for 30 s, and 72 °C for 30 s. The final extension step was performed at 72 °C for 5 min. The PCR products were sequenced. Sequence homology was evaluated using nucleotide BLAST (BLASTN, NCBI).

The analysis of sequenced data and sequenced similarity searches of nucleotide and protein were performed using the BLAST (N) program of NCBI database and DNAMAN software. Function prediction and structure analysis of csn SH21 were performed using NCBI and Uniprot database (http://www.uniprot.org/). The phylogenetic analysis indicating the relationship of csn SH21 to other chitosanases was constructed with MEGA 6. The secondary structure and homology model was built by using Phyre2 server (http://www.sbg.bio.ic.ac.uk/phyre2/html/page.cgi).

### 4.10. Statistical Analyses

Each experiment was performed at least in triplicate. The test was used to analyze the differences among mean values at the 95% confidence level. All the statistical analyses were performed using the Statistica 6.0 software package.

## 5. Conclusions

In this study, chitosanase SH21, which inhibited the growth of *F. solani*, was identified in *B. subtilis* SH21 and purified. SEM and TEM revealed that this antifungal protein appeared to inhibit *F. solani* by both causing hyphal ablation, distortion, or abnormalities, and cell wall depression. Moreover, chitosanase SH21 had a typical a-helix structure, belonging to the GH46 family. These results provide a theoretical basis for a topical cream against fungal infections in agriculture and a selection marker on fungi. Further studies are necessary to better understand the mechanisms underlying the inhibitory effects of chitosanase SH21.

## Figures and Tables

**Table 1:** Protein yield of each purification step.

Purification Procedure	Volume (mL)	Total Protein (mg)	Yield (%)
Crude enzyme	1000	260	100
(NH_4_)_2_SO_4_ ppt	15	125	48.1
Superdex75PG	25	37.5	14.4
SP-HPR	5	3	1.2

**Table 2:** The results of amino acid identification.

Protein IDs	Protein Names	Sequence Coverage (%)	Mol. Weight (kDa)	Score
D4FZ80	Chitosanase	86.3	30.72	323.31

## Sample Availability

The *Bacillus subtilis* SH21 is available from the authors.
